# Information Needs During Pregnancy and Its Associated Factors in Afghan Pregnant Migrant Women in Iran

**DOI:** 10.1177/2150132720905949

**Published:** 2020-02-18

**Authors:** Mahnaz Sharifi, Leila Amiri-Farahani, Shima Haghani, Syedeh Batool Hasanpoor-Azghady

**Affiliations:** 1 Department of Reproductive Health and Midwifery, Faculty of Nursing and Midwifery, Iran University of Medical Sciences, Tehran, Iran; 2 Department of Reproductive Health and Midwifery, Nursing Care Research Center (NCRC), School of Nursing and Midwifery, Iran University of Medical Sciences, Tehran, Iran; 3 Department of Biostatistics, Nursing Care Research Center, Iran University of Medical Sciences, Tehran, Iran

**Keywords:** information needs, information seeking, pregnant women, migrants, Afghan

## Abstract

**Background:** Access to pregnancy-related information is an important requirement for all pregnant women, especially women at risk, such as immigrants. Regarding this, the present study was conducted to determine the information needs during pregnancy and its associated factors in the Afghan pregnant women. **Methods:** This cross-sectional study was conducted on 280 Afghan pregnant women who received care at the prenatal clinics of selected health care centers in the southeast of Tehran in 2018. The study population was selected using the continuous sampling method. The sampling was performed through the continuous sampling method from all the Afghan pregnant women who received care at the prenatal health centers of the southeast of Tehran. **Results:** Among the information needs during pregnancy, the fetal (83.34 ± 20.65) and smoking (62.61 ± 28.88) domains had the highest and lowest mean scores by percentage, respectively. The information needs during pregnancy showed a statistically significant relationship with age, women’s education level, husband’s education level, duration of living in Iran, place of residence, insurance status, number of children, place of the previous delivery, and routine prenatal care. Based on the multiple regression model, only the place of birth and place of residence accounted for 19% of information needs during pregnancy. **Conclusion:** As the findings indicated, the prenatal care–related education should address the domains that are unknown for Afghan women. Furthermore, in this education, the demographic and reproductive characteristics of the recipients should be taken into account to improve the pregnancy outcome among this population.

## Introduction

Pregnancy or the phenomenon of becoming a parent is a major life-changing event and it is considered one of the most sensitive periods of women’s life^1^ during which they require special care and information.^2^ Women experience multiple emotional and physical alterations during pregnancy. Therefore, prenatal training and care services should address this wide range of physical and emotional changes^3^; women feel insufficient information regarding pregnancy and newborn care.^4^

The women living in developing countries or less knowledgeable communities have a limited role in the selection of their health care time, location, and even approach.^5^ Also, limited knowledge in terms of pregnancy and its associated risk factors leads to referral delay, thereby increasing childbirth complications.^6^ The majority of women in developing countries have usually a low education level. Along with low education, they have insufficient information concerning childbirth.^7^

On the other hand, insufficient information about pregnancy is accompanied by more frequent referrals to the doctor, increased emotional complications, and augmented costs of paraclinical and diagnostic tests implemented to eliminate the concerns during pregnancy.^6,8^ In this regard, the provided information would have a higher impact if tailored to women’s requirements and interests.^9^ When mothers meet their information needs, it might enhance their satisfaction with the childcare process and result in the improvement of care quality.^10^

The mentioned issues also apply to immigrant Afghan women as an active fertile population in Iran. Three million Afghan refugees live in Iran. In terms of gender, 44% of Afghan immigrants are female. According to the United Nations High Commissioner for Refugees, Afghan population in Iran suffer from such problems as poverty, malnutrition, serious health and hygiene issues, and low level of information and knowledge.^11^

Based on several studies around the world, immigrant, and refugee women have higher rates of stillbirth, intrauterine death, sudden infant death syndrome, and perinatal mortality, compared with the females of the host society.^12,13^ Furthermore, immigrants are more prone to danger and harm due to their different living, cultural, and social conditions; accordingly, they are vulnerable.^14^

The immigrant Afghan women in Australia reported that they need to receive more information during pregnancy and labor. Complications can be decreased through the improvement of information in this population.^15^

Health care providers have limited time for providing the required information for mothers during pregnancy. It has been seen that health care providers are not comprehensively aware of the fundamental needs of mothers. Therefore, they try to make decisions and give information based on their previous experience learned by other mothers. It can be concluded that this approach might cause the suggestion of misleading information, which is incapable of satisfying essential requirements. However, addressing the interests and information needs of these women can encourage this group to accept the information and properly apply them in their lives.^16^ There are some recognized barriers for Afghans when they are seeking health care. First, most of Afghans in Iran are poor and do not have insurance, so they cannot pay for some clinical investment like sonography or laboratory testing. Second, a large number of them are living in the outskirts and do not have access to health care or are not aware of the health care system around them. Another barrier against their health care access is culture and language. Iranians and Afghans indeed speak Persian but the Afghan Persian is Dary, which is different to some extent to Iranian language. This diversity in culture and language make some misunderstanding for them when they seek health care system.

There are limited data regarding the pregnancy information needs of Afghan women. Moreover, the review of the literature revealed no study evaluating these needs in Afghan women. With this background, the present study aimed to determine the information needs during pregnancy and its associated factors in the Afghan pregnant women who received care at the prenatal health centers located in the southeast of Tehran, Iran, in 2018.

## Materials and Methods

This cross-sectional study was performed on 280 Afghan pregnant women who received care at the prenatal health centers of the southeast of Tehran in 2018. The sample size required for evaluating the information needs of Afghan women regarding reproductive health was estimated at 280 individuals with an accuracy of *d* = 0.2 and 95% confidence level.

According to the report of immigration and passport police head office in Tehran, most of the Afghans live in the southeast of Tehran. The southeast of Tehran consists of 4 parts that were easier for sampling. Data collection was performed by the selection of 100, 80, 60, and 60 samples from Varamin, Pishva, Pakdasht, and Gharchak, respectively. Sampling was carried out during the office hours of the health centers on all days of the week during June 2018 to August 2018. The sampling was performed through the continuous sampling method from all the Afghan pregnant women who received care at the prenatal health centers of the southeast of Tehran. The inclusion criteria were as follows: being Afghan and having a pregnancy file in the prenatal center clinic. On the other hand, the participants with more than 10% skipped responses were excluded from the study.

The data collection tools included a demographic and reproductive characteristics form and a questionnaire investigating reproductive health information needs. This questionnaire consisted of 37 items in 10 domains, namely physical health (2 items), mental health (4 items), nutrition (6 items), physical activity (4 items), medicine consumption (5 items), maternal care (6 items), fetal care (2 items), smoking (4 items), management of minor complications (2 items), and management of major complications (2 items).^1,3,6,17^

Each item in this questionnaire was scored on a 5-point Likert-type scale (0 = no idea, 1 = not important, 2 = a little important, 3 = important, 4 = very important). The score of each domain was calculated separately, and the overall score was obtained by summing up the scores of all domains. The minimum and maximum scores of this instrument were 0 and 148, respectively. Given the difference in the number of items in each domain, the score for each domain was reported by percentage to facilitate homogeneity and comparison.

The items of the questionnaire were compiled based on literature review, evaluation of the various tools addressing reproductive health information needs, and viewpoints of several reproductive health specialists. Both face and content validities were applied to validate the tool for assessing pregnancy information needs. For the determination of face validity, face-to-face interviews were held with 30 Afghan women (they were eligible for entry to study) that to evaluate the level of difficulty and ambiguity of the questionnaire. Regarding the content validity, the viewpoints of 9 faculty members of the Department of Midwifery of Iran University of Medical Sciences, Tehran and 3 obstetric specialists from Afghanistan were applied. Also, the reliability of this tool was confirmed using the internal consistency method rendering a Cronbach’s alpha coefficient of .96. The questionnaires were filled out by implementing interviews.

### Statistical Analysis

The data were analyzed in SPSS software, version 21. The demographic and reproductive characteristics data were presented as frequency distribution, central tendency, and index of dispersion (ie, mean and standard deviation). The relationship between the pregnancy information needs and demographic data was examined by the independent-sample *t* test and 1-way analysis of variance. Also, the linear regression model with backward elimination method was used to estimate the effect of each independent variable (ie, demographic characteristics) on the dependent variable (ie, pregnancy information needs).

Moreover, all variables showing a significant change (*P* < .05) were entered into the multivariate linear regression model using the backward elimination method. Before the implementation of multivariate analysis, the regression assumptions, such as normality of the residuals, homoscedasticity, multicollinearity, and independence of the residuals, were checked.

### Ethical Considerations

The present research was approved by the Research Deputy of Iran University of Medical Sciences after obtaining the ethical code of IR.IUMS.REC1396, 9513593001 from the Ethics Committee of the university. Furthermore, informed written consent was obtained from the participants, and the respondents were completely informed of the study purpose and procedures. Also, they were assured of the confidentiality of information.

## Results

According to the results, the mean age of the participants was 26.9 ± 6 years. The mean total score of information needs during pregnancy was 115.46 ± 23.77 among the population. For comparative purposes, the mean score of each domain of the questionnaire was calculated by percentage. The mean scores of the domains of the questionnaire, including fetal care, management of minor complications, management of major complications, use of medication, maternal care, physical health, nutrition, physical activity, emotional activity, and smoking were 83.34%, 83.08%, 82.36%, 81.89%, 81.83%, 80.17%, 77.79%, 76.38%, 76.36%, and 62.61%, respectively. Among the different domains of the questionnaire, fetal (83.3%) and smoking (62.6%) domains had the highest and lowest mean scores, respectively (Table 1). Tables 2 and 3 present more information based on demographic and reproductive characteristics and their relationship with pregnancy information needs.

**Table 1.** Mean Scores of Pregnancy Information Needs Among the Afghan Women Who Received Care at the Prenatal Clinic of Selected Health Care Centers in the Southeast of Tehran, Iran, in 2018 (n = 280).

Pregnancy Information Needs	Total Score			
	Mean	SD	Maximum	Minimum
Physical health	6.41	1.90	8	0
Physical health (%)	80.17	23.85	100	0
Mental health	12.21	3.72	16	0
Mental health (%)	76.36	23.30	100	0
Nutrition	18.67	3.97	24	2
Nutrition (%)	77.79	20.72	100	8.33
Physical activity	12.22	3.43	16	0
Physical activity (%)	76.38	21.48	100	0
Medication intake	16.37	3.57	20	4
Medication intake (%)	81.89	18.87	100	20
Maternal care	19.63	3.93	24	5
Maternal care (%)	81.83	16.38	200	20.83
Fetal care	6.66	1.65	8	0
Fetal care (%)	83.34	20.65	100	0
Smoking	10.01	4.62	16	0
Smoking (%)	62.61	28.88	100	0
Management of minor complications	6.64	1.71	8	0
Management of minor complications (%)	83.08	21.40	100	0
Management of major complications	6.58	1.80	8	0
Management of major complications (%)	82.36	22.52	100	0
Total score	115.46	23.77	148	25
Total score (%)	78.01	16.06	100	16.89

**Table 2.** Relationship Between Pregnancy Information Needs and Demographic Characteristics Among the Afghan Pregnant Women Who Received Care at the Prenatal Clinics of Selected Health Centers in the Southeast of Tehran, Iran, in 2018 (n = 280).

Variable		n (%)	*P* ^a^	Variable		n (%)	*P* ^a^
Age (years)	<19	28 (10)	**.02**	Time of residence in Iran (years)	<5	56 (20)	**.02**
	19-25	87 (31.1)			5-10	20 (7.1)	
	26-30	92 (32.9)			11-15	16 (5.7)	
	31-35	46 (16.4)			16-20	78 (27.9)	
	>35	27 (9.6)			>20	110 (39.3)	
Woman’s occupation	Housewife	264 (94.3)	.98	Religion	Shia	208 (74.3)	.43
	Employed	16 (5.7)			Sunni	72 (25.7)	
Woman’s education level	Illiterate	84 (30)	**<.001**	Ethnicity	Hazara	186 (66.4)	.56
	Elementary	75 (26.8)			Pashtun	14 (5)	
	Junior high school	69 (24.6)			Tajik	60 (24.1)	
	Senior high school	42 (15)			Others	20 (7.1)	
	Academic	10 (3.6)		Income level (Toman)	<1 000 000	84 (30)	.18
Marital status	Married	277 (98.9)	.56		1 000 000-1 400 000	88 (31.5)	
	Divorced	2 (0. 7)			1 500 000-2 000 000	94 (33.7)	
	Widow	1 (0.4)			>2 000 000	13 (4.7)	
Spousal occupation^b^	Laborer	205 (73.5)	.24	Insurance	Yes	34 (12.1)	**.04**
	Self-employed	74 (26.5)			No	246 (87.9)	
Place of residence	Urban	217 (77.5)	**.03**	Spousal education level^b^	Illiterate	70 (25.1)	**.003**
					Elementary	92 (33)	
	Rural	63 (22.5)			Junior high school	75 (26.9)	
Place of birth	Iran	110 (39.28)	**.001**		Senior high school	31 (11.1)	
	Afghanistan	170 (60.71)			Academic	11 (3.9)	

a Boldfaced values indicate statistical significance.

b There was a case of spousal death.

**Table 3.** Relationship between pregnancy information needs and reproductive characteristics data of the Afghan women who received care at the prenatal clinics of selected healthcare centres in the Southeast of Tehran, Iran, 2018 (n=280).

Variable		Frequency (%)	*P* ^a^	Variable		Frequency (%)	*P* ^a^
Gestational age (weeks)	<15	40 (14.3)	.70	Previous delivery	Caesarean	41 (14.6)	.40
	15-28	123 (43.9)			Vaginal	144 (51.4)	
	>28	117 (41.8)			None	95 (33.9)	
History of abortion	Yes	48 (17.1)	.30	Place of the previous delivery	Home	33 (17.8)	**<.001**
	No	232 (82.9)			Public hospital	145 (78.4)	
Number of children	None	95 (33.9)	**.03**		Private hospital	7 (3.8)	
	1	126 (45)		Previous ill child	Yes	14 (7.6)	.58
	2	36 (12.8)			No	171 (92.4)	
	3	23 (8.2)		Maternal illness	Yes	44 (15.7)	.42
Routine care	Yes	212 (75.7)	**.009**		No	236 (84.3)	
	No	68 (24.3)		Participation in childbirth classes	Yes	47 (16.8)	.12
Visit duration	Very short	4 (1.4)	.18		No	233 (83.3)	
	Short	33 (11.8)		Maternal drug intake	Yes	44 (15.71)	.42
	Moderate	181 (64.6)					
	Long	53 (18.9)			Yes	236 (84.28)	
	Very long	9 (3.2)					

a Boldfaced values indicate statistical significance.

After investigating the relationship of each variable with pregnancy information needs, those showing a significant relationship were included in the multivariate linear regression model using the backward elimination method. Among the relevant variables, the place of residence and place of birth remained in the model. According to the results, the women residing in rural areas had −8.23 times lower mean pregnancy information need than urban residents. Also, the women with a history of delivery in a public hospital had −7.71 times lower mean pregnancy information need, compared to those with a history of delivery in a private hospital. Accordingly, the place of residence and place of delivery as independent variables could account for 19% of the variation in variables dependent on the mean information need during pregnancy (Table 4).

**Table 4.** Multivariate Linear Regression Analysis Examining the Effect of Demographic and Reproductive Characteristics on the Mean Score of Information Needs During Pregnancy Among the Afghan Women Who Received Care at the Prenatal Clinics of Selected Health Care Centers in the Southeast of Tehran, Iran, in 2018 (n = 280).

Independent Variable		*B* Coefficient	Standardized Coefficient	Statistics	*P* ^a^	Confidence Interval	*R* ^2^
Age		−0.61	−0.15	−0.158	.11	−1.3 to 0.14	.19
Duration of living in Iran		0.04	0.01	0.21	.82	−3.1 to 3.9	
Number of living children		0.39	0.02	0.21	.82	−0.34 to 0.43	
Education level	Illiterate	−17.47	−0.33	1.85	.06	−36.02 to 1.06	
	Elementary	−7.63	−0.14	−0.83	.40	−25.6 to 10.3	
	Junior high school	−7.70	−0.14	−0.85	.39	−25.3 to 9.9	
	Senior high school	−6.66	−0.10	−0.73	.46	−24.5 to 11.2	
	Academic	Reference category					
Spousal education level	Illiterate	−3.93	−0.07	−0.43	.66	−21.6 to 13.7	
	Elementary	2.52	0.05	0.29	.77	−14.4 to 19.4	
	Junior high school	2.28	0.04	0.26	.79	−14.6 to 19.1	
	Senior high school	1.69	0.02	0.19	.84	−15.2 to 18.6	
	Academic	Reference category					
Place of previous delivery	Home	−3.32	−0.04	−0.58	.56	−14.5 to 7.2	
	Private hospital	−7.71	0.16	2.01	**.04**	0.16–15.2	
	Public hospital	Reference category					
Insurance status	No	−3.54	−0.04	−0.80	.42	−12.2 to 5.1	
	Yes	Reference category					
Place of residence	Rural	−8.23	−0.14	−2.48	**.01**	−14.7 to −1.7	
	Urban	Reference category					
Place of birth	Iran	3.41	0.07	0.94	.34	−3.6 to 10.5	
	Afghanistan	Reference category					

a Boldfaced values indicate statistical significance.

## Discussion

The present study is the first attempt investigating the information needs of Afghan women during pregnancy in Iran. The results of the current study showed the fetal and smoking domains had the highest and lowest means scores, respectively.

Regarding the fetal care domain, the mean scores of information regarding the diagnosis of fetal genetic diseases and the stages of fetal development were 3.36 and 3.3, respectively. In a study conducted by Almalik and Mosleh^1^ on 150 pregnant women in Jordan to assess information needs, the mean score of information on fetal development during pregnancy was reported as 3.37. Therefore, there is a consistency between the mean information need obtained by Afghan and Jordan women.

Kahouei et al^6^ conducted a study to investigate the information needs of pregnant women in Semnan, Iran. The results showed that two-thirds of women required information on fetal genetic screening tests, while about half of Afghan women considered this information as an important domain.^6^ This difference is attributed to the difference in the education level of the participants of the two studies. In this regard, 9.5% of the respondents of the mentioned study were illiterate, whereas the illiteracy rate in the current study was 30%.

In another study, Kamali et al^18^ evaluated the information needs of pregnant women in Kerman, Iran. In the mentioned study, the highest level of information required was related to fetal care (86%).^18^ Similarly, in the current study, the fetal care domain had the highest mean score; however, the scores obtained for this domain differ between the 2 studies. These differences can be ascribed to the high education level of the women in the study conducted by Kamali et al.^18^ However, in the current study, 3.6% of Afghan women had an academic degree.

In a study carried out by Bjelk et al,^19^ more than two-thirds of women required information about the stages of fetal development. On the other hand; half of the Afghan women reported the importance of this domain. The lower levels of information needs among Afghan women, compared with Swedish ones in the study by Bjelk et al^19^ can be attributed to the differences in the level of education of the women participating in the 2 studies. In comparing the results, 54% and 6.3% of Swedish and Afghan women had university degrees, respectively. According to the literature, there is a direct association between the level of education and seeking information about fetal health.^20^

In the present study, the management of minor complications was ranked as the second most important domain after fetal care. In this regard, 53.6% of the women reported this domain as very important. However, in the study by Kahouei et al,^6^ information regarding sleep disorders, nausea/vomiting, and back pain was important for 10.5%, 39%, and 29% of the women, respectively. The observed difference in the results of the two studies is probably due to the lower education level of Afghan women and their younger age at their first pregnancy. According to Kelly et al,^20^ the enhancement of education level and aging people achieve more experience and skills to cope with their physical problems.

In the current study, the mean score of information on the management of major complications during pregnancy was obtained as 3.31 ± 0.92. This item ranked the third most important domain of pregnancy information needs in Afghan women. The mean score of this domain in Jordanian women was estimated at 3.39 ± 0.95.^1^ In Kahouei et al’s study^6^; about half of the women ranked information about the sign risk of pregnancy as very important. However, in the current study, more than half of the Afghan women rated this item as the most important domain.

In a study performed by Kamali et al,^18^ 61.5% of pregnant women required information about the major complications of pregnancy. Despite the similarities between the results of the present study and those obtained by Kahouei and Almalik, the scores of information needs were higher in the study by Kamali et.al. This is because one of the inclusion criteria adopted by Kamali et al^18^ was academic education, while in the present study the illiterate women were not excluded from the investigation process.

Medicine intake was the fourth most important information needs domain among the Afghan women. The mean score of information about the benefits of taking medications and supplements for fetus and mother was estimated at 3.41 ± 0.81. In a study conducted by Almalik and Mosleh,^1^ the mean scores of this variable for fetus and mother were obtained as 3.43 ± 0.8 and 3.45 ± 0.8, respectively. In the study conducted by Kahouei et al,^6^ 39.5% of the pregnant women required information about the vitamins and minerals during pregnancy. However, half of Afghan women regarded this item as very important. Accordingly, the lower levels of the social and economic status of the womenled them to seek more information in this regard.^21^

Maternal care was ranked as the fifth most important information domain during pregnancy. In this regard, the mean scores of information about the cost of care and breast care methods during pregnancy were 3.44 ± 0.88 and 3.7 ± 1.9, respectively. The obtained results are consistent with the findings of the study performed by Almalik and Mosleh.^1^

The physical domain (80.17 ± 23.85) was ranked as the sixth information needs of Afghan women during pregnancy. In this regard, 51.9% and 58.9% of the women required information on normal and abnormal physiological changes during pregnancy, respectively. The results of the study by Kahouei et al^6^ showed that 57% and 52% of women reported the need for information about normal physiological changes and risk factors as their priority. Our obtained results in terms of this domain are in line with those reported by Almalik and Mosleh.^1^

The mean score of nutrition was obtained as 79.77 ± 20.72; therefore, it was ranked as the seventh information required by Afghan women. In the study conducted by Kahouei et al,^6^ the availability of information about the effect of food on fetal intelligence and beauty was given the priority by more than a quarter of pregnant women; however, this rate was estimated at 65% in the current study.

The domain of physical activity was ranked the eighth information needs of Afghan women. In this regard, the means scores of forbidden physical activities and traveling during pregnancy were 3.23 ± 0.95 and 2.62 ± 1.30, respectively. The obtained results revealed that Jordan and Afghan women had similar mean values regarding the allowed levels of physical activities.^1^ In the study by Kahouei et al,^6^ information about traveling and sexual activities during pregnancy was the primary concern of 18% and 50.5% of the women, respectively.

In the present study, 33.6% and 53.9% of the Afghan women paid great attention to travel and sexual activities during pregnancy, respectively. On the other hand, in a study conducted by Singh et al,^17^ it was revealed that 23% of women were eager to gain information about sexual activities during pregnancy. Moreover, Singh et al^17^ mentioned that recent improvements in national health care services and different training classes on issues related to pregnant women have raised women’s awareness and decreased their information needs.

Moreover, Kamali et al^18^ found that 62.5% and 68% of women required information about exercise and sexual relationship during pregnancy, respectively. The participants of the mentioned study required more information about the sexual relationship than the subjects of the present study.

The emotion was ranked as the ninth information domain the Afghan women needed. In the study by Kahouei et al,^6^ information regarding domestic violence and its effect on pregnant women was the main concern in 57.5% of women. However, in the current study, it was important for 55.4% of Afghan women to gain information about domestic violence and its effect on pregnant women.

The study by Kahouei et al^6^ revealed that information about depression and stress was the main concern of 27% and 62.5% of women, respectively. However, these rates were 50.4% and 46.1% for the Afghan women. This means that immigrant women need more knowledge, compared with the local women of society.^1^

Smoking was considered as the least important information domain during pregnancy. In the present study, Afghan women had a higher mean score in this domain, compared with participants in the study by Almalik and Mosleh.^1^ Moreover, in the study by Singh et al,^17^ only 6% of women tended to have more information about smoking.

Kahouei et al^6^ reported that the need for information about smoking and its effects on fetal health was the priority of 12% of women. Similar to this study, the information about smoking obtained the least importance in the studies by Kahouie et al^6^ and Singh et al.^17^ The lack of enthusiasm for Afghan women to seek more information about smoking might be attributed to the lower level of smoking among women than among men.^22^

The mean score of information needs during pregnancy was significantly associated with age, women’s education level, husbands’ education level, duration of living in Iran, place of residence, place of birth, insurance status, number of children, place of the previous delivery, and routine prenatal care. The results of this study are consistent with those obtained by Singh et al.^17^ This difference can be explained by the younger age of women in this study, compared with those investigated by Singh et al who were older than 35 years. Kamali et al^18^ reported a statistically significant association between the mean of information needs during pregnancy and variables, such as age, education, and occupation. However, their results are not consistent with the findings of this study regarding the occupation status.

In the study conducted by Kamali et al,^18^ the participants were housewives (52%), university students (13%), and employees (30%). Nonetheless, in the current study, 94.4% and 5.6% of Afghan women were housewives and employed, respectively. Accordingly, this explains the lack of a significant relationship between occupational status and the pregnancy information need in this study.

Sanginian^23^ conducted a study on the information needs of women during pregnancy in Norway and Iran. In the mentioned study, information needs during pregnancy showed no statistically significant association with age and education level.^23^ Furthermore, Adelkhah and Olszewska^24^ carried out a study titled “Iranian Afghans” to evaluate the life of Afghans in Iran. They stated that with the increased length of stay in Iran, many immigrants considered education as a necessary skill for women and men and an opportunity to get a better social status.^24^ Therefore, Afghan women who were born in Iran had a higher chance of going to school and gaining access to education than those born in Afghanistan. This explains the reason that people with higher levels of education are more likely to seek more information, compared with illiterate ones.^25^

In the study carried out by Kamali et al,^18^ the mean of information needs had a significant association with the number of previous pregnancies and children, as well as gestational age. The results of the mentioned study in terms of the number of children are in line with our results. However, they are inconsistent with our results regarding the number of previous pregnancies and gestational age. The reason for the discrepancy between the 2 studies can be due to the difference in the number of women without children in the 2 studies. In the current study, 33.9% of the women had no child, whereas, in the mentioned study, 56.5% of the participants were childless.^18^

The place of delivery was identified as one of the predictors of the pregnancy information needs of Afghan women. Women with a history of delivery in a private hospital reported lower information needs than those with previous deliveries in a public hospital or at home. The place of residence was another predictive variable for the information need during pregnancy. Also, Afghan women who live in cities required more pregnancy-related information than those in rural areas. No study has investigated the effect of the place of residence and delivery on the information need during pregnancy. This study has some limitations. A large number of Afghan women were illiterate or had a low education level. Therefore, for questionnaire completion, a color spectrum rather than a Likert-type scale was utilized. Future studies are recommended to investigate the information needs during pregnancy from both women’s and health care providers’ points of view. Also, the information needs of women in different domains were investigated in this study without any concerns about information resources. Accordingly, it is suggested that future studies be conducted to review the information resources of Afghan pregnant women.

## Conclusion

The findings of the present study revealed the higher needs of Afghan women for all domains of pregnancy information, compared to the similar rates reported in the studies conducted across the world. As a result, it is necessary to provide information about prenatal care while considering the demographic and reproductive characteristics of those who need these services. The suggestions of this study for policymakers in Iran or international organizations are to provide equal facilities to teach Afghan women how to read and write, insurance for all of them, and some staff who are completely familiar with Afghans culture in the health care center. These steps would be beneficial for both staff and refugees.
